# Serum levels of vitamin D, parathyroid hormone and calcium in relation to survival following breast cancer

**DOI:** 10.1007/s10552-014-0413-3

**Published:** 2014-06-22

**Authors:** Linnea Huss, Salma Butt, Signe Borgquist, Martin Almquist, Johan Malm, Jonas Manjer

**Affiliations:** 1 Department of Surgery, Skåne University Hospital, Lund University, 205 02 Malmö, Sweden; 2 Department of Oncology, Skåne University Hospital, Lund, Sweden; 3 Division of Oncology, Department of Clinical Sciences, Lund University, Lund, Sweden; 4 Section for Clinical Chemistry, Department of Laboratory Medicine, Skåne University Hospital, Lund University, Malmö, Sweden; 5 Department of Plastic Surgery, Skåne University Hospital, Lund University, Malmö, Sweden; 6 Department of Surgery, Skåne University Hospital, Lund University, Lund, Sweden

**Keywords:** Breast cancer, Calcium, Mortality, PTH, Survival, Vitamin D

## Abstract

**Purpose:**

Vitamin D, parathyroid hormone (PTH) and calcium in blood are correlated with each other. Previous studies have suggested vitamin D to have anti-proliferative effects on tumor cells, whereas PTH may have carcinogenic effects. A cancer disease may influence calcium levels in blood, but less is known about calcium and its potential effect on cancer risk and survival. The aim of this study was to examine pre-diagnostic levels of vitamin D (25OHD), PTH and calcium in relation to survival after breast cancer.

**Methods:**

The Malmö Diet and Cancer Study enrolled 17,035 women between 1991 and 1996. 672 patients developed incident invasive breast cancer up until 31 December 2006. Serum samples collected at baseline were analyzed for 25OHD, PTH and calcium. All patients were followed until 31 December 2010 using the Swedish Cause of Death Registry. The analytes were divided into tertiles and the risk of death from breast cancer was analyzed using an adjusted Cox proportional hazards analysis, yielding hazard ratios with 95 % confidence intervals.

**Results:**

Levels of 25OHD and breast cancer mortality were associated in a u-shaped manner with the highest mortality among patients in the first (2.46: 1.38–4.37) and third tertiles (1.99: 1.14–3.49), as compared to the second. An inverse relation was found between calcium levels and breast cancer mortality, with the lowest mortality in the third tertile, (0.53: 0.30–0.92) as compared to the first. There was no clear association between PTH and breast cancer mortality.

**Conclusions:**

This study shows that pre-diagnostic 25OHD and calcium may affect survival following breast cancer.

## Introduction

Vitamin D has been suggested to have anti-proliferative effects on breast tumor cells in animal and in vitro studies [1]. Ecological studies have shown a difference in breast cancer incidence and survival related to geography, suggesting a beneficial effect of vitamin D levels due to solar exposure [2–4] and others have shown a better survival in patients diagnosed with breast cancer during summer and autumn [5, 6].

Several prospective epidemiological studies have investigated the relationship between vitamin D and breast cancer incidence, but with conflicting results [1, 7–9]. A recent meta-analysis of studies which had measured vitamin D levels (25OHD in serum or plasma) close to diagnosis in early stage breast cancer, found an association between low levels of vitamin D and a high mortality, i.e., a poor survival [10]. Only one study has previously investigated pre-diagnostic serum levels of vitamin D in relation to breast cancer mortality among breast cancer patients. Freedman et al. [11] found an inverse relationship between low pre-diagnostic levels of vitamin D (25OHD) and high breast cancer mortality.

Levels of vitamin D and parathyroid hormone (PTH) are inversely correlated with each other [12], and both influence the level of calcium in blood. PTH is secreted from the parathyroid gland when calcium levels are low and stimulates release of calcium from bone into blood and synthesis of active vitamin D (1,25(OH)_2_D) from its storage form (25OHD). It has been suggested in experimental studies that PTH has a carcinogenic and tumor promoting effect [13–16], and it has also been indicated that primary hyperparathyroidism may increase the risk of breast cancer [17–20]. To our knowledge, no previous study has investigated the relationship between levels of PTH and breast cancer survival.

It is well known that calcium levels may be increased following different cancer forms. One previous study has shown an increased incidence of breast cancer with high pre-diagnostic calcium levels [21]. It is, however, unknown what impact pre-diagnostic calcium levels may have on breast cancer survival. Since calcium has been shown to be an important intracellular messenger, involved in proliferation, apoptosis and cell signaling [22], it is possible to hypothesize that calcium may affect survival following breast cancer.

In 1991, inclusion in a population-based prospective cohort study began in Malmö, creating The Malmö Diet and Cancer Study (MDCS), including 17,035 women. Blood samples taken at baseline are now available for analysis.

Our main hypothesis is that low vitamin D (25OHD) is associated with a poor survival following breast cancer, i.e., a high mortality among cases. Our secondary hypothesis is that high pre-diagnostic PTH is also associated with a poor survival. As a third explorative analysis, we investigated if calcium also had an effect on survival.

## Materials and methods

### The Malmö Diet and Cancer Study (MDCS)

Between 1991 and 1996, all residents in the Southern Swedish city of Malmö, born 1923–1950, were invited to participate in a population-based prospective cohort study. A total of 41 % of eligible subjects participated and 17,035 women completed the baseline examination [23]. Written informed consent was obtained from all participants. Baseline examination included a dietary assessment and a self-administered questionnaire on different life-style factors. Moreover, a trained nurse performed anthropometric measurements and blood samples were drawn. Subjects were included, and had their blood samples drawn, evenly over the calendar year, except for less recruitment in December and June, and none in July. The ethical committee in Lund, Sweden, approved the MDCS (LU 51-90), and the present study (Dnr 652/2005 and Dnr 23/2007).

### Study population

For identification of breast cancer cases within the MDCS cohort, The Swedish Cancer Registry was used. Prior to baseline examination, 576 out of the 17,035 women were diagnosed with breast cancer. These women were categorized as prevalent breast cancer cases and therefore excluded from the current analysis. In all, 766 women were diagnosed with breast cancer up until December 31, 2006, but two incident cases had not donated blood at baseline [7]. We found that 77 tumors were cases of ductal cancer in situ, and these cases were excluded as the present study intended to examine survival, i.e., there is a very low mortality, if any, associated with in situ breast cancer. Another 15 cases were excluded due to bilateral cancer, as it was difficult to interpret information about tumor characteristics in these cases. Finally, a total of 672 women with invasive unilateral breast cancer were included in the present analysis. Mean time from baseline examinations to diagnosis was 7.12 years with a standard deviation (SD) of 3.82.

### Clinical information

Information on type of surgery and planned adjuvant therapy, recommended by a treatment conference immediately following surgery, was retrieved from clinical notes. Some 41 % (*n* = 272) underwent mastectomy, and 57 % (*n* = 382) had a partial mastectomy. Lymph nodes were examined after sentinel node biopsy in 26 % (*n* = 173) and after axillary dissection in 62 % (*n* = 416). Adjuvant treatment with radiotherapy was planned for 54 % (*n* = 361) of patients, endocrine treatment in 46 % (*n* = 312), and 14 % (*n* = 92) were planned for chemotherapy, Table 1.

**Table 1:** Vital status in relation to age at baseline

Factor	Survival			
	Alive (*n* = 517)	Dead from breast cancer (*n* = 101)	Dead from other cause (*n* = 54)	Total (*n* = 672)
	Number (column percent) Mean (SD) in italics			
Age at baseline	*56.3 (6.8)*	*58.3 (8.0)*	*61.8 (6.8)*	*57.0 (7.2)*
Age at diagnosis	*63.7 (7.6)*	*64.6 (8.9)*	*67.8 (7.4)*	*64.2 (7.9)*
Size				
1–10 mm	159 (30.8)	3 (3.0)	17 (31.5)	179 (26.6)
11–20 mm	240 (46.6)	38 (37.6)	18 (33.3)	297 (44.2)
≥21 mm	110 (21.5)	52 (51.5)	18 (33.3)	181 (26.9)
Unknown	6 (1.2)	8 (7.9)	1 (1.9)	15 (2.2)
Lymph node status				
Positive	128 (25.1)	63 (62.4)	11 (20.4)	204 (30.4)
Negative	384 (74.3)	36 (35.6)	42 (77.8)	462 (68.8)
Unknown	3 (0.6)	2 (2.0)	1 (1.9)	6 (0.9)
Distant metastasis				
Yes	0 (0.0)	9 (8.9)	0 (0.0)	9 (1.3)
No	514 (99.8)	91 (90.1)	54 (100.0)	661 (98.4)
Unknown	1 (0.2)	1 (1.0)	0 (0.0)	2 (0.3)
Nottingham grade				
I	150 (29.1)	8 (7.9)	16 (29.6)	175 (26.0)
II	244 (47.4)	31 (30.7)	20 (37.0)	296 (44.0)
III	90 (17.5)	48 (47.5)	14 (25.9)	152 (22.6)
Unknown	31 (6.0)	14 (13.9)	4 (7.4)	49 (7.3)
Histological type				
Ductal	345 (66.9)	66 (65.3)	33 (61.1)	445 (66.2)
Lobular	96 (18.8)	21 (20.8)	9 (16.7)	127 (18.9)
Other/mixed	47 (9.1)	2 (2.0)	9 (16.7)	58 (8.6)
Unknown	27 (5.2)	12 (11.9)	3 (5.6)	42 (6.2)
ER				
0–10 % pos	47 (9.1)	18 (17.8)	6 (11.1)	71 (10.6)
>10 % pos	380 (73.9)	63 (62.4)	43 (79.6)	488 (72.6)
Unknown	88 (17.0)	20 (19.8)	5 (9.3)	113 (16.8)
PgR				
0–10 % pos	183 (35.4)	50 (49.5)	28 (51.9)	261 (38.8)
>10 % pos	195 (37.9)	24 (23.8)	16 (29.6)	236 (35.1)
Unknown	137 (26.7)	27 (26.7)	10 (18.5)	175 (26.0)

Age at diagnosis and prognostic factors for breast cancer

### Histopathological analysis

Information on laterality, tumor size and lymph node metastasis was retrieved from medical records and histopathological reports. All invasive tumors, diagnosed between 1991 and 2004, were pathologically re-evaluated by one senior pathologist. The re-evaluation concerned tumor invasiveness, tumor type according to WHO and grading according to Elston–Ellis [24, 25]. Tumors diagnosed between 2005 and 2006 were classified according to WHO type and Elston–Ellis grade at diagnosis, thus information was readily available from clinical notes and pathology reports. Estrogen receptor status (ER) and progesterone receptor status (PgR) on all tumors were evaluated with tissue microarray technique (TMA), using immunohistochemical (IHC) analysis with specific antibodies as described in detail previously [25]. In line with Swedish clinical practice, the cutoff points for dichotomizing tumors, as being negative or positive, were 0–10 and 11–100 % positive nuclei, respectively.

### Laboratory analysis

At baseline examination, serum was extracted within 1 h from venipuncture and samples were thereafter stored at −80 °C [26]. Serum from identified cases of breast cancer was retrieved from the MDCS bio bank and analyzed for 25OHD, PTH and calcium. The samples had not been previously thawed. High-pressure liquid chromatography (HPLC) was used to analyze 25OHD_3_, and laboratory analysis was successful in 655 out of 672 cases in the present study population. PTH was analyzed with the Immunolite^®^ 2000 Intact PTH immunoassay (Diagnostic Products corporation, Los Angeles, CA), 664 successful analyses. Total calcium was successfully analyzed in 661 cases by neutral carrier ion-selective electrode [27]. Unsuccessful analyses were due to inadequate volume or quality of sera. The analysis of blood samples was performed during 2007 as part of a previous case–control study and has previously been described in detail [7].

### Endpoint retrieval

The Swedish Cause of Death Registry was used to identify cases that had deceased as well as their cause and date of death. End of follow-up was the date of death, date of emigration or December 31, 2010. Mean time from diagnosis until end of follow-up was 8.7 years (SD: 4.0). Subsequently, the women were divided into three different groups: (1) women still alive at end of follow-up; (2) women with breast cancer as cause of death or with breast cancer as a contributing cause of death which were classified as “dead from breast cancer,” i.e., “breast cancer-specific mortality”; and (3) women deceased from causes unrelated to breast cancer (“dead from other cause”).

### Statistical methods

Levels of 25OHD, PTH and calcium were divided into tertiles. Survival was assessed as mortality from breast cancer per 100,000 person-years. In order to test differences in mortality between tertiles, a Cox’s proportional hazards analysis, yielding hazard ratios (HR) and 95 % confidence intervals (CI), was used. The assumption of proportional hazards was met as tested by log—minus log plots.

The model was subsequently adjusted for factors known to influence levels of 25OHD, PTH and calcium, such as season of blood draw and age at baseline. In order to adjust for storage time, year of baseline examination was included in the model.

The Cox analysis was further adjusted for factors known to affect survival following breast cancer such as age at diagnosis, tumor size, lymph node status, the presence of distant metastases, Nottingham grade, histological type, ER status and PgR status. All confounders were tested one at a time in the model in order to see which factor affected hazard ratio (HR) the most.

As a sensitivity analysis, we adjusted our exposures for each other. As an example, 25OHD was adjusted for the other exposures, PTH and calcium, individually and combined.

To assess the risk of reverse causality, we performed sensitivity analyses, repeating all analyses, excluding women diagnosed with breast cancer within 2 years from the baseline examination (*n* = 82). Another sensitivity analysis, in which the Cox analyses were also adjusted for different types of adjuvant therapy, was also performed.

Moreover, we made sensitivity analyses in which we stratified the analyses for premenopausal, respectively, postmenopausal women at the time of diagnosis, and for body mass index (BMI) <25 (considered normal weight) versus BMI ≥25 (overweight).

## Results

Out of 672 women, 101 had died due to breast cancer, which gives a breast cancer-specific mortality of 1,738/100,000 person-years. A comparison between different outcomes and factors possibly affecting survival is presented in Table 1. Table 2 shows the distribution of treatment in different groups defined the outcome.

**Table 2:** Vital status in relation to treatment

Factor	Vital status			
	Alive (*n* = 517)	Dead breast cancer (*n* = 101)	Dead other cause (*n* = 54)	Total (*n* = 672)
	Column percent			
Surgical treatment				
Mastectomy	36.6	60.4	40.7	40.5
Partial mastectomy	61.7	31.7	57.4	56.8
Local excision or surgical biopsy	0.4	0.0	0.0	0.3
Unknown	1.4	7.9	1.9	2.4
Axillary surgery				
No axillary dissection	9.9	5.9	20.4	10.1
Axillary dissection	58.6	75.2	68.5	61.9
Sentinel node biopsy	29.8	13.9	9.3	25.7
Singular node biopsy	0.4	0.0	0.0	0.3
Unknown	1.4	5.0	1.9	1.9
Planned adjuvant radiotherapy				
No	36.0	35.6	53.7	37.4
Yes	53.4	61.4	42.6	53.7
Unknown	10.6	3.0	3.7	8.9
Planned adjuvant endocrine therapy				
No	51.5	43.6	63.0	51.2
Yes	48.5	56.5	37.1	48.8
Anti-estrogen	37.9	43.6	29.6	38.1
Aromatase inhibitor	3.5	7.9	1.9	4.0
Other/unknown drug	4.6	2.0	5.6	4.3
Unknown	2.5	3.0	0.0	2.4
Planned adjuvant chemotherapy				
No	78.3	63.4	90.7	77.1
Yes	10.6	33.7	5.6	13.7
Unknown	11.0	3.0	3.7	9.2

There was a statistically significantly higher breast cancer-specific mortality (HR) among patients in the first 1.84 (1.08–3.13) as well as the third 1.81 (1.06–3.07) tertile of 25OHD as compared to the second tertile (Table 3). In the adjusted analysis, these associations were strengthened, factors that affected the results most were lymph node status and distant metastasis, and remained statistically significant.

**Table 3:** Tertiles of 25OHD_3_, PTH and Ca in relation to breast cancer mortality

Analyte	Tertile	Range	Numbers within tertile	Person-years	Dead breast cancer	Breast cancer mortality/100,000	HR^a^	HR^b^	HR^c^
25OHD_3_ (nmol/L)	1	≤75	221	1,879	39	2,076	1.84 (1.08–3.13)	2.04 (1.19–3.51)	2.46 (1.38–4.37)
	2	76–99	216	1,863	21	1,127	1	1	1
	3	≥100	218	1,911	39	2,041	1.81 (1.06–3.07)	1.78 (1.05–3.04)	1.99 (1.14–3.49)
							*p* trend: 0.95	*p* trend: 0.62	*p* trend: 0.51
PTH (pmol/L)	1	≤2.95	223	1,842	36	1,954	1.31 (0.81–2.12)	1.32 (0.81–2.14)	0.98 (0.53–1.65)
	2	2.96–4.41	220	2,000	30	1,500	1	1	1
	3	≥4.42	221	1,904	34	1,786	1.20 (0.73–1.96)	1.21 (0.74–1.98)	0.86 (0.51–1.47)
							*p* trend: 0.71	*p* trend: 0.70	*p* trend: 0.62
Calcium (mmol/L)	1	≤2.36	235	2,067	38	1,838	1	1	1
	2	2.37–2.43	224	1,932	33	1,707	0.93 (0.58–1.48)	0.90 (0.56–1.44)	0.80 (0.48–1.34)
	3	≥2.44	202	1,727	28	1,622	0.88 (0.54–1.44)	0.82 (0.50–1.34)	0.53 (0.30–0.92)
							*p* trend: 0.61	*p* trend: 0.40	*p* trend: 0.02

^a^Crude analysis

^b^Adjusted for season and year of blood sample, and age at baseline

^c^Adjusted for same factors as ^b^ but also for age at diagnosis, size of tumor, Elston–Ellis grade, histological type, ER status, PGR status, lymph node status and distant metastasis at diagnosis

When studying pre-diagnostic levels of PTH, there was also a higher breast cancer-specific mortality among subjects in the first 1.31 (0.81–2.12) as well as in the third 1.20 (0.73–1.96) tertile (Table 3); however, the results were not statistically significant. Several factors coincided to make the associations disappear in the adjusted analysis, but when adjusting for size of tumor and lymph node status, the reduction was strongest (data not shown).

An inverse relationship was seen between calcium levels and breast cancer-specific mortality, with lower mortality among subjects within the third tertile 0.88 (0.54–1.44). This association was not statistically significant in the crude analysis, but it was stronger and turned statistically significant 0.53 (0.30–0.92) when the analysis was adjusted for known prognostic factors (Table 3). Factors adding most to the stronger association were lymph node status and distant metastasis.

In the adjusted analyses, where our studied exposures were adjusted for the other exposures, all results remained the same (data not shown).

In order to exclude women with subclinical breast cancer at the time of baseline blood donation, we repeated analyses excluding women diagnosed with breast cancer within 2 years as a sensitivity analysis. We found similar associations regarding 25OHD in the first tertile (2.22: 1.20–4.11), but statistical significance was lost for the third tertile (1.65: 0.89–3.06). No association could be seen between PTH levels and breast cancer mortality in this analysis. There was still a low mortality from breast cancer among patients with calcium levels within the third tertile in the adjusted analysis, but this association did not reach statistical significance (0.69: 0.37–1.30).

The sensitivity analysis adjusting also for adjuvant therapy showed similar associations in all analyses (data not shown).

Due to statistical instability in the premenopausal group (*n* = 65), the stratified analyses on menopausal status showed inconclusive results. In the postmenopausal group (*n* = 587), results were similar in all analyses, though confidence intervals were somewhat widened (data not shown).

In the analyses, where we stratified for BMI, we noticed that results were attenuated in the group with BMI ≥25 (*n* = 326), compared to the group with BMI <25 (*n* = 346), though this observation is inconclusive due to low statistical power in the analysis (data not shown).

## Discussion

We found a statistically significant u-shaped relationship, between pre-diagnostic levels of 25OHD and breast cancer-specific mortality, with higher mortality, i.e., poor survival, among patients with 25OHD levels within the first and third tertiles as compared to the second. There was no association between PTH and breast cancer-specific mortality. Regarding levels of calcium and breast cancer-specific mortality, we saw that patients within the highest tertile had a lower mortality, i.e., a better survival.

### Vitamin D and breast cancer

The finding that low 25OHD levels were associated with a high breast cancer-specific mortality is in line with our hypothesis. It is now well known that vitamin D inhibits growth of tumor-derived cells from breast [28] and promote apoptosis in breast cancer cells [29]. On a molecular level, active vitamin D (1,25(OH)_2_D) has been shown to act as a cancer inhibitor in many other ways, such as enhanced DNA repair, immunomodulation and protection against antioxidants, although there are areas not yet understood [30, 31]. These mechanisms may indeed explain the results seen in the present study.

One previous study has shown a better survival for breast-, colon- and prostate cancer patients diagnosed during summer or early fall, which would indicate advantages of adequate vitamin D levels during treatment [6]. It has also been suggested that less favorable outcome for African–American women with breast cancer in the USA is due to lower habitual vitamin D status among these women [32]. Directly measured sufficient levels at diagnosis in early breast cancer have in other studies been shown to improve survival [10, 33–37]. Experimental and epidemiological studies taken together; they are consistent with our finding that lower pre-diagnostic levels of 25OHD are related to poor survival. One study measuring vitamin D before diagnosis found an inverse relationship between pre-diagnostic levels of vitamin D (25OHD) and breast cancer mortality [11]. When analyzing breast cancer mortality, they choose to stratify 25OHD in two categories with levels >62.5 and ≤62.5 nmol/L [11], hence they differ only between high and low 25OHD. This cut point is within our first tertile and is therefore also in line with our results.

To this date, there are no available guidelines regarding adequate 25OHD levels, though the Committee of the Institute of Medicine has recommended 40–50 nmol/liter as a lower acceptable level and that levels above 125 nmol/L should raise concern among clinicians in North America [38]. These recommended clinical cut points are within the first, respectively, third of our tertiles, and 5.9 % (*n* = 39) of our subjects had levels underneath 50 nmol/L, 9.3 % (*n* = 62) above 125 nmol/L.

In addition to the findings that women with low levels of 25OHD had a high mortality, i.e., a poorer survival, we found that patients within the third tertile were also at a higher risk for a breast cancer-related death. To date, this has not been shown previously and the findings do not support our primary hypothesis. However, a similar pattern, with a u-shaped relationship, between levels of 25OHD and risk of subsequent prostate cancer has been found in our cohort [39]. Also, one previous study on 25OHD levels measured at diagnosis and overall mortality in postmenopausal breast cancer patients, showed the lowest mortality among patients within the second tertile [37]. Since most of the anti-carcinogenic effects of vitamin D seem to be mediated by the vitamin D receptor (VDR) [30], individual- or tumor-specific differences in VDR may be of importance.

### PTH and breast cancer

We did not find any association between levels of PTH and breast cancer-specific mortality. Previous experimental studies have suggested that PTH may be associated with poor breast cancer survival due to carcinogenic and tumor promoting effects [13–16], such as regulating angiogenesis and osteoclastogenesis in bone metastasis by breast cancer cells [40]. Therefore, our hypothesis was that there would be an association. Due to intra-individual variation of PTH levels [41, 42], there is a risk of misclassification, which might have affected our results. There are no previous results reported from epidemiological studies on PTH and breast cancer survival, and our explorative analysis is the first within the area.

### Calcium and breast cancer

High levels of calcium at diagnosis of breast cancer have previously been associated with large tumors, and this may well be an effect of the tumor *per se* [43]. Previous findings suggest an increased incidence and more aggressive breast cancer tumor characteristics, associated with higher pre-diagnostic calcium levels [7, 21, 44]. In contrast, our explorative analysis found an association between high pre-diagnostic levels of calcium and a lower breast cancer-specific mortality. This finding needs further scientific attention, and the expression or activity of the calcium receptor may modulate the effect of calcium on breast tumors.

### Methodological issues

This study was performed using blood samples taken before diagnosis. Therefore, the tumor itself cannot have influenced the analyzed levels. The sensitivity analysis excluding women diagnosed within 2 years from baseline showed similar results regarding 25OHD and mortality from breast cancer, but statistical significance was lost in the upper tertile, assumingly due to loss of statistical power with a decreasing number of outcomes, more precisely 94 deaths from breast cancer instead of 113.

It must be considered that there is only one blood sample available for analysis, sometimes taken many years before diagnosis, and it is possible that this sample does not reflect the individuals’ habitual vitamin D, PTH and calcium status. Previous studies have shown, though, that 25OHD measured at two times, several years apart have a high correlation [45, 46].

Regarding PTH, it has been shown that there is a short-term (up to 6 weeks) intra-individual variation of about 25 % [41, 42]. PTH also shows a relatively large circadian fluctuation [41], and the time of the day for blood donation in the present study has not been recorded [7]. Therefore, there is a risk of misclassification of PTH levels that may have attenuated a potential possibly obscure true association between pre-diagnostic levels of PTH and mortality from breast cancer. Contrary to PTH, total serum calcium has been shown to have a low intra-individual variation over short as well as long time [47, 48].

Vitamin D levels tend to decrease with increasing age, whereas PTH and calcium increases [49, 50]. Therefore, we adjusted our analyses for age. We decided not to adjust for menopause in our analysis, as menopause is heavily dependent on age. In a sensitivity analysis, where we stratified for menopause, the analysis showed similar associations for postmenopausal women, but the analysis was unstable regarding premenopausal women, due to small numbers (*n* = 65) in this group.

Another factor that is known to affect serum levels of 25(OH)D is season [51], but as this factor was included in the multivariate analysis, we consider that such variation ought to have affected our results only to a minor extent.

Since all Swedish residents are given a unique civil registration number, it is possible to link all women to different registries. The Swedish Cause of Death Registry that was used to retrieve information on cause of death, had a coverage of 97.3 % in 2008 [52], and it has been shown to be correct in 90 % of cases where malignant tumors is the cause of death [53]. Therefore, it is expected that data concerning cause of death is complete and correct to a great extent.

As it has been previously shown that women within the MDCS cohort have a higher incidence of breast cancer, but lower breast cancer mortality, than non-participants, there is a risk of a selection bias. The mortality risk between participants in this study group and general population of Malmö, hence, may differ. However, as there was a broad distribution of 25OHD, and to some extent PTH in our material, we consider that relative risks were less likely to have been affected by a potential selection bias.

Primarily, we chose not to adjust for BMI in this study, since previous studies have shown that a high BMI is associated with low levels of vitamin D [54], and high BMI is also associated with a poor prognosis [55]; hence, it may be part of the casual pathway. A previous study has suggested the possibility of trapping vitamin D in subcutaneous fat [56]. Therefore, an adjustment for BMI could possibly have masked an association between vitamin D levels and breast cancer prognosis. When we stratified the analyses for normal versus overweight, we noted that associations were stronger in the overweight group compared to normal weight, which could be explained by the above reasoning.

Since the analysis was adjusted for prognostic factors that are used when deciding on adjuvant treatment, we chose to present the analyses not adjusting for treatment as this may have lead to an over-adjustment. Moreover, the sensitivity analysis that included treatment showed similar results as the main analysis.

Apart from the variables adjusted for in the present analyses, there may have been other factors of interest, such as information on human epidermal growth factor receptor 2 (HER-2), which is a known prognostic factor that influences both choice of treatment as well as prognosis of a diagnosed breast cancer. Unfortunately many tumors in our material were diagnosed before HER-2 was recognized and used in clinical practice.

Fortunately, breast cancer-specific mortality is relatively low, but this means few deaths from breast cancer in the present study. Nonetheless, we were able to find statistically significant data to support that pre-diagnostic 25OHD levels influence mortality from breast cancer. In this analysis, we could not find statistically significant associations between pre-diagnostic levels of PTH and breast cancer survival and also the association between levels of calcium and breast cancer mortality was weak. Since outcome, i.e., deaths from breast cancer are sparse, there is a possibility of type II error and true associations could have been missed.

## Conclusion

Women with pre-diagnostic 25OHD levels within the first and third tertiles as compared to the second have a higher breast cancer-specific mortality, i.e., a poor survival. There was no association between pre-diagnostic levels of PTH and breast cancer mortality. A weak association was seen between high levels of pre-diagnostic calcium and low breast cancer mortality. Our analysis suggests that vitamin D levels may affect breast cancer survival, but that both low and relatively high levels may have an adverse effect.

## Appendix

See Table 4.

**Table 4:** Tertiles of vitamin D3 in relation to age and breast cancer characteristics

Factor	Tertile of D3				
	1st ≤ 75 (*n* = 221)	2nd 76–99 (*n* = 216)	3rd ≥ 100 (*n* = 218)	Unknown (*n* = 17)	Total (*n* = 672)
	Column percent (mean and SD in italics)				
Age at diagnosis (years)					
Mean	*63.6*	*64.9*	*63.8*	*67.0*	*64.2*
SD	*7.8*	*7.8*	*8.0*	*8.8*	*7.9*
Size					
1–10 mm	24	29	28	18	27
11–20 mm	48	42	42	59	44
≥21 mm	27	26	28	24	27
Unknown	2	3	2	0	2
Lymph node status					
Positive	28	31	32	35	30
Negative	71	68	67	65	69
Unknown	1	1	1	0	1
Distant metastasis					
Yes	1	1	2	0	1
No	99	99	98	100	98
Unknown	1	1	0	0	0
Nottingham grade					
I	25	30	22	47	26
II	43	43	46	41	44
III	24	20	25	6	23
Unknown	8	7	7	6	7
Histological type					
Ductal	68	67	63	71	66
Lobular	19	17	21	24	19
Other/mixed	6	12	9	0	9
Unknown	7	5	7	6	6
ER					
0–10 % pos	12	8	12	6	11
>10 % pos	72	74	73	71	73
Unknown	16	18	16	24	17
PgR					
0–10 % pos	43	33	40	35	39
>10 % pos	33	39	33	41	35
Unknown	24	28	27	24	26
